# Longitudinal Study of Thyroid Hormones between Conventional and Organic Farmers in Thailand

**DOI:** 10.3390/toxics8040082

**Published:** 2020-10-05

**Authors:** Noppanun Nankongnab, Pornpimol Kongtip, Nichcha Kallayanatham, Ritthirong Pundee, Jutharak Yimsabai, Susan Woskie

**Affiliations:** 1 Department of Occupational Health and Safety, Faculty of Public Health, Mahidol University, 420/1 Rajvidhi Road, Phayathai, Rajthavee, Bangkok 10400, Thailand; pornpimol.kon@mahidol.ac.th (P.K.); nichcha.kal@gmail.com (N.K.); 2 Center of Excellence on Environmental Health and Toxicology, EHT, Bangkok 10400, Thailand; 3 Mahidol University, Nakhonsawan Campus, Nakhon Sawan 60130, Thailand; rtg.pun@gmail.com; 4 Department of Medical Technology and Clinical Pathology, Buddhachinaraj Phitsanulok Hospital, 90 Sithamma Traipidok Road, Muang, Phitsanulok 65000, Thailand; jutharak@gmail.com; 5 Department of Public Health, University of Massachusetts Lowell, One University Ave, Lowell, MA 01854-2867, USA; Susan_Woskie@uml.edu

**Keywords:** Thyroid hormones, conventional farmers, organic farmers, longitudinal study

## Abstract

Many pesticides are endocrine-disrupting chemicals that can interfere with hormone levels. This study aimed to assess the longitudinal impact of exposure to pesticides on thyroid hormone levels, including Thyroid Stimulating Hormone (TSH), Free Triiodothyronine (FT3), Free Thyroxine (FT4), Triiodothyronine (T3), and Thyroxine (T4). Both conventional (i.e., pesticide using) and organic farmers were interviewed using questionnaires, and blood samples were collected at 7–9 a.m. to determine thyroid hormone levels for four rounds, with a duration of eight months between each round. A linear mixed model of the natural log of the individual hormone levels used random intercepts for subjects while controlling gender, baseline age, and body mass index (BMI) was used to compare between conventional and organic farmers or the impact of cumulative days of spraying insecticides, herbicides or fungicides. The estimated marginal means of the thyroid hormone levels (TSH, FT3, T3, and T4) estimated from the linear mixed models were significantly higher among the conventional farmers than organic farmers. As cumulative spray days of insecticide, herbicide or fungicide increased, TSH and FT3 increased significantly. FT4 decreased significantly as cumulative spray days of insecticide or fungicide increased. These findings suggest that the insecticides, herbicides, and fungicides sprayed by conventional farmers exert endocrine-disrupting activities, altering the hypothalamic-pituitary-thyroid (HPT) axis homeostasis.

## 1. Introduction

Many pesticides have been identified as endocrine-disrupting chemicals (EDCs). At low doses, EDCs may interfere with the synthesis, transport, metabolism, and elimination of hormones, leading to decreased hormone levels [1]. In animal studies, thyroid hormone production can be inhibited by some pesticides, including amitrole, cyhalothrin, fipronil, ioxynil, maneb, mancozeb, pentachloronitro-benzene, prodiamine, pyrimethanil, thiazopyr, ziram, and zineb [2]. In humans, thyroid hormones are essential for the development of the brain, inner ear, eye, heart, kidneys, bone, skeletal muscle, and regulation of energy metabolism [1]. Pesticide levels in blood or urine during the early development of children have been linked to thyroid dysfunction [3], an increase in hypothyroidism [4,5], and brain function impairment [6]. An increased risk of hypothyroidism was associated with ever-use of the herbicides 2,4-D, 2,4,5-T, 2,4,5-TP, alachlor, dicamba, and petroleum oil and the insecticides chlordane, Dichlorodiphenyltrichloroethane (DDT), heptachlor, lindane, and toxaphene, diazinon, malathion, and carbofuran [7]. Current knowledge on the impact of pesticides on human thyroid function is still limited [7,8,9]. The extensive work of the U.S. Agricultural Health Study reported that exposure to organophosphate insecticides in agricultural spouses was a risk factor for developing several hormonally-related cancers, including thyroid, breast, ovary cancers, and lymphoma [10]. However, limited studies have investigated the impact of pesticides on human thyroid functions [1,11]. Therefore, the objective of this work was to conduct a longitudinal study by following conventional and organic farmers for two years. Data were collected for four rounds (eight months per round) to evaluate the changes in thyroid hormone levels, including Thyroid Stimulating Hormone (TSH), Free Triiodothyronine (FT3), Free Thyroxine (FT4), Triiodothyronine (T3), and Thyroxine (T4).

## 2. Materials and Methods 

### 2.1. Study Population

The recruitment of subjects was carried out with the assistance of health-promoting hospitals, public health volunteers, and community leaders. Conventional farmers comprised rice farmers, vegetable farmers, and sugarcane farmers. Rice farmers and vegetable farmers were recruited from Wat Phrik and Bungphra, sub-districts of Phitsanulok Province. Sugarcane farmers were recruited from Kaotong, sub-district of Nakorn Sawan Province. Organic farmers were recruited from five sub-districts in Yasothorn Province, including Kammaet, Nong Yor, and Na So (sub-district in Kut Chum district), Sang Ming (sub-district in Loeng Nok Tha district), and Bak Ruea (sub-district in Maha Chana Chai district). The organic farmers mainly grew rice, together with vegetables and fruits. The inclusion criteria included male or female farmers over 18 years old who did not self-report having diabetes, high blood pressure, thyroid, or heart disease. For conventional farmers, one farmer who sprayed pesticides was selected from each household. Organic farmers were obligated to utilize certified organic practices for all of their crops. One organic farmer was selected from each household. There were 213 conventional farmers and 225 organic farmers in the first round. After eight months, the study participants in the second round were reduced to 199 (conventional farmers) and 222 (organic farmers), with response rates of 93.43% and 98.67%, respectively. The response rate of conventional farmers and organic farmers was 92.96% and 97.75%, in the third round and 96.22% and 92.63%, respectively, in the fourth round. This study was approved by the Ethical Review Committee for Human Research, Faculty of Public Health, Mahidol University (Approval No. MUPH 2015-146, date of approval: 28 August 2015). Informed consent was obtained from all participants.

### 2.2. Data Collection

The subjects were recruited in February to April 2016. The baseline physical health examination was conducted for the first, second, third, and fourth rounds in November to December 2016, July 2017, and April to May 2018, and January to February 2019, respectively. The questionnaires comprised farmer characteristics, self-reported stress, home and demographic information, self-reported health problems, and agricultural activities. History of self-reported pesticide use (days of spraying) for the prior eight months was collected for each crop cycle for each pesticide type. Stress symptoms in the past two to four weeks, including sleeping difficulty, reduced concentration, irritation, boredom, or not wanting to meet people, were used. Data on weight, height, waist circumference, body composition (Body composition analyzer; Tanita model DC-360, Amsterdam, Netherland), and blood pressure (taken twice every 10 min apart and were averaged) were collected. Body mass index (BMI) was calculated as weight (kg)/height (m)^2^. Blood samples were collected in the morning from 7 to 9 a.m. Sera extracted from blood samples in non-heparinized vacutainer tubes were stored at −20 °C until analysis. All samples were analyzed at Buddhachinaraj Hospital, the regional medical center in Phitsanulok province, using standard clinical laboratory methods. The analysis of TSH, T3, T4, FT3, and FT4 was conducted using a paramagnetic particle, chemiluminescent immunoassay in human serum (UniCel DxI 800 Access Immunoassay System, Beckman Coulter, Atlanta, GA, USA) [12]. The limit of detection was 0.005 μIU/mL for TSH, 0.09 ng/dL for FT3, 0.15 ng/dL for FT4, 0.10 ng/mL for T3 and 0.50 μg/dL for T4.

### 2.3. Statistical Analysis

Descriptive analysis of the demographic characteristics and clinical outcomes (thyroid hormone level, BMI) was done using Chi-Square, Fisher Exact test, and Mann-Whitney U test using SPSS (Version 18; PASW Statistics Base 18, Serial no. 5082368, ID no. 5071846). Individuals who met the inclusion criteria and came for at least one round of testing were included in the analysis. Since the thyroid hormone levels were not normally distributed, we used the natural log for all analyses. For each thyroid hormone, we used a linear mixed model (LMM) with subject-specific random intercepts. The models were adjusted a priori for age and gender because the incidence of thyroid disease in women is 5–20 times higher than in men, increasing with age [13]. Additional covariates that were considered for inclusion based on significance and/or change in exposure parameters of >10% included BMI, education, income, current smoking, current alcohol use, stress symptoms, insecticide use at home, second job, and marital status. Of these, only BMI was added as a covariate in the final models based on our criteria for covariate inclusion. For exposure, we used either the organic or conventional farmers’ categorization or the self-reported cumulative days of spraying insecticides, herbicides, or fungicides for each consecutive round.

## 3. Results

### 3.1. General Characteristics

Table 1 shows the general characteristics of the study participants. The average age and education of organic farmers were significantly higher than those of conventional farmers. The percentage of study participants having a BMI lower than 18.49 kg/m^2^ was 8.7%. The subjects tended to have a higher end of abnormal BMI (≥25 kg/m^2^). Conventional and organic farmers having highly abnormal BMI accounted for 42.5% and 25.8%, respectively. A significantly higher BMI was observed with conventional farmers. Compared to organic farmers, conventional farmers were more likely to be male farmers, current drinkers, and current smokers. Organic farmers had a higher percentage of second jobs than conventional farmers.

### 3.2. Comparison between Conventional and Organic Farmers

The marginal geometric means estimated from the repeated measures linear mixed models for all four rounds of data collection showed that conventional farmers had significantly higher TSH, FT3, T3, and T4 levels than organic farmers (Table 2). Table 3 shows the geometric means estimated from the linear regression models for each round of data collection. Data indicated that the conventional farmers had significantly higher TSH levels than organic farmers for every round. In round one, round three, and round four, the higher FT3 and T3 levels were observed with the conventional farmers compared to the organic farmers. The conventional farmers had significantly higher T4 levels than organic farmers in round three and round four. On the other hand, the organic farmers had significantly higher FT4 levels than the conventional farmers in round three.

### 3.3. Cumulative Pesticide Spraying Day of Conventional Farmers

Conventional farmers used various pesticides, including insecticides, herbicides, and fungicides in their rice, vegetable, and sugarcane fields. Biomarker collections were conducted for four rounds; the duration of each round was eight months. The cumulative numbers of self-reported spray day of each type of pesticides (insecticides, herbicides, and fungicides) were calculated from four rounds. Table 4 shows the cumulative self-reported pesticide spraying days (mean (SD) and median (IQR)) for each round. The mean numbers of cumulative spraying days of insecticides were higher than those of herbicides and fungicides for all rounds. For fungicides, the percentage of conventional farmers who did not use fungicides in round one, round two, round three, and round four were responsible for 51.6%, 45.2%, 41.1%, and 40.4%, respectively.

### 3.4. Percentage Change of Thyroid Hormones per 10 Days of Cumulative Pesticide Spraying

The percentage change in thyroid hormone levels per 10 cumulative days of pesticide spraying is presented in Table 5. TSH and FT3 were found to increase significantly (2.92% and 0.95%, respectively), and FT4 was found to decrease significantly (−0.43%) for each 10 cumulative days of insecticide spraying. For herbicides, TSH, and FT3 were found to increase significantly (4.68% and 1.72%, respectively) for each 10 cumulative days of spraying. TSH and FT3 increased significantly (3.28% and 0.64%, respectively), and FT4 was found to decrease significantly (−0.58%) for each 10 cumulative days of fungicide spraying.

## 4. Discussion

In our previous cross-sectional study, which reported only the results from the round one baseline data collection, we found that conventional farmers had significantly higher TSH, FT3, and T3 levels compared to organic farmers [12]. In this current longitudinal study, we followed up the conventional and organic farmers for three years with four rounds of thyroid hormone measurement (eight months per each round). We still found that conventional farmers had significantly higher TSH, FT3, T3, and T4 levels than organic farmers, even when accounting for our repeated measurements (Table 2). Unlike in our baseline cross-sectional study, we also found the T4 levels of conventional farmers were significantly higher than those of the organic farmers. When examining the dose-response relationship with pesticides, we found a significant increase in TSH and FT3 with increasing cumulative use of insecticides, herbicides, and fungicides, and a significant decrease in FT4 with increasing cumulative use of insecticides and fungicides.

In our previous cross-sectional study of the baseline round one data, we were able to collect a record of the amount of each type of pesticide sprayed in the previous eight months and estimated the moles of active ingredient applied [12]. As the study progressed over three years, farmers were unwilling to diligently collect this data, so we were unable to reliably estimate the concentration of each type of pesticide applied for each pesticide type. In the baseline cross-sectional study, we found some preliminary evidence that the number of moles of herbicide applied in the previous eight months was significantly associated with a small increase in TSH levels. When looked at specific pesticides, we found small but significant increases in TSH, FT3, and T3 hormone levels from the use of paraquat as well as impacts on other hormone levels by diuron, atrazine, acetochlor and glyphosate and a group of ‘other’ herbicides including alachlor, propanil, and butachlor [12]. In this study, we found that changes in hormone levels were associated with higher cumulative spray days of insecticides, herbicides, and fungicides. However, it should be noted that most farmers used both insecticides and herbicides, while a fewer number also used fungicides. In round one (51.6%), round two (45.2%), round three (41.1%), and round four (40.4%) of the conventional farmers reported they did not use fungicides (Table 4). The top three insecticides used by conventional farmers were cypermethrin (29.6%), abmectin (28.6%), and chlorpyrifos (16.0%). For herbicides, the top three were glyphosate (69.2%), paraquat (66.2%), and 2,4-D (61.5%). The major three fungicides commonly used by the conventional farmers included difenoconazole (14.8%), propineb (10.8%), and propiconazole (8.9%).

Regarding insecticides, animal studies of organophosphate insecticide exposures have reported decreased T3 and T4, but increased TSH levels in rats [14,15]. Long-term exposure to chlorpyrifos-methyl induced hypothyroidism (reduced T4 and increased TSH) in the offspring of Sprague-Dawley rats [16]. In fish, cypermethrin exposure was found to decrease the T3 and T4 levels, while TSH levels increased [17]. It should be noted that animal studies tend to use higher insecticide doses compared to the doses human are normally exposed to. Also, the studies did not use mixed pesticide exposures nor account for the biological differences inherent in the human species [18,19]. In human studies, the urinary 3,5,6-trichloro-2-pyridinol (TCPY), metabolite of chlorpyrifos and chlorpyrifos methyl was found to have a positive association with T4 levels and negative association with TSH levels in male participants. Conversely, TCPY was positively associated with TSH levels in females for participants over 60 years old [20]. Lacasaña et al. [21] found that increasing urinary DAP (Dialkyl phosphate) levels were associated with increased TSH and T4 in male floriculture workers. Meeker, et al. [22] found that for male participants of reproductive age, urinary TCPY levels were positively associated with TSH and negatively associated with FT4 levels. Farokhi and Taravati [23] reported that pesticide sprayers exposed to organophosphate and organochlorine pesticides had significantly increased TSH and decreased T3 compared to controls. In this study, we found that an increase in cumulative days of spraying insecticides significantly increased TSH and FT3, and decreased FT4 levels. The inconsistency between some results in the previous studies and our study could be explained by differences in the pesticides used, the sample size, the race/ethnicity of workers, and related genetic polymorphisms involved in the metabolism of pesticides, or different methods, and timing of exposure/biomarker measurement. In this cohort, the conventional farmers reported using insecticides, including chlorpyrifos, methyl parathion, carbosulfan, cypermethrin, carbofuran, abamectin, profenofos, and others.

For herbicides, animal studies have found that exposure to 2,4 D decreases T3 and T4 levels and increased hypothyroidism in rats [24]. Several chlorinated herbicides, dinitrophenols, fenoprop, linuron, bromoxynil, 2,4,5-trichlorophenoxyacetic acid, trichloroacetic acid, dichlorophenol, and trichlorophenol reduced T4 levels through interference with transport proteins [25]. In this current study, we found the cumulative days of spraying herbicides was associated with an increase in TSH and FT3, but we did not see an association between cumulative herbicide spray days and reduced T4. The herbicides we reported used in our baseline cross-sectional study included glyphosate, paraquat, 2,4 D, diuron, acetochlor, ametrin, atrazine, alachlor, propanil, butachlor, etc. [12]. In humans, Goldner et al. [9] found a significant association between reports of ever using 2,4 D and hypothyroidism in male private pesticide applicators. Goldner et al. [7] also observed a significant association between ever using alachlor and hyperthyroidism. Shrestha et al. [5] found an increase in self-reported incident hypothyroidism in pesticide applicators who reported ever using 2,4 D. Goldner et al. [9] observed a significant association between ever using paraquat and self-reported hypothyroidism among women in the U.S. Agricultural Health Study (AHS). Tsatsakis et al. [26] reported that postmortem analysis of humans with paraquat poisoning revealed detectable amounts of paraquat in the thyroid gland. They suggested that the thyroid could be susceptible to the effects of paraquat.

For fungicides, ethylene bisdithiocarbamates (EBDC) are widely used for the protection of fruits, vegetables, and field crops from fungal diseases. The main degradation product of the EBDC (e.g., maneb, mancozeb, ziram, and zineb) is ethylene thiourea (ETU) [11]. In animal studies, higher exposure levels of ETU, mancozeb, and maneb decreased T3 and T4 [27,28]. Rats exposed to mancozeb had reduced T4 levels [29]. However, if exposed to tebuconazole fungicide, they had significantly reduced T3 [30]. Zebrafish larvae exposed to both hexaconazole and tebuconazole fungicides had decreased T4 and T3 levels [31]. Carbendazim, a widely used broad-spectrum benzimidazole fungicide, was found to cause histopathological damage to the thyroid and increased serum T3 levels in rats [32]. The amitrole (herbicide), ethylenethiourea (fungicide), mancozeb (fungicide), soy isoflavones, and benzophenone 2 were found to inhibit the production of thyroperoxidase (TPO) enzyme and prevented the synthesis of thyroglobulin, leading to decreased T3 and T4 [33]. In this current study, we found cumulative spray days of fungicide exposure significantly increased TSH, FT3, and reduced FT4 levels. The fungicides used by farmers in this study were propiconazole, difenoconazole, azoxystrobin, propineb, chlorothalonil, tricyclazole, hexaconazole, carbendazim, mancozeb, metalaxyl, and others. Piccoli et al. [34] reported that total lifetime use years of use of herbicides, fungicides, and the insecticide dithiocarbamate was associated with increased TSH and decreased FT4 in men. The report of ever using the organochlorine chlordane, the fungicides benomyl or maneb/mancozeb, or the herbicide paraquat were significantly associated with hypothyroidism (increased TSH and decreased T4). Maneb/mancozeb is the only fungicide that has been associated with both hyperthyroidism and hypothyroidism [9].

The main limitation of the current study was the pesticide information came from self-reports during interviews with the farmers at each round and could be affected by recall bias. In addition, because farmers used multiple types of pesticides, it is difficult to sort out the relative contributions of these highly correlated exposures. The subjects were asked whether they were diagnosed or had received treatment for diabetes, high blood pressure, or heart disease. If so, they were excluded from this study; this could have produced some recruitment bias. Thyroid hormone levels depend on iodine intake levels and iodine levels in the blood. However, iodine intake levels and blood iodine levels of the participants were not determined in this study. Finally, before becoming organic farmers, the organic farmers in this study had used pesticides for 0 to 40 years (an average of 16 years). The average time of pesticide use for the conventional farmers in this study was 26.9 years (ranging from 4 to 51 years). Thus, the differences between organic and conventional farmers may be underestimated.

The main strength of this study is that it reported a repeated measures longitudinal examination representing four rounds of data collection (eight-month period for each round) over two years. Thus, the findings represent robust estimates of the impact of farming type (organic versus conventional) and the number of cumulative spray days of pesticide use.

## 5. Conclusions

We found significantly increased TSH, T3, T4, and FT3 among conventional farmers compared to the organic farmers after controlling for other covariates. In addition, higher cumulative days of spraying insecticides, herbicides, and fungicides were significantly associated with increased TSH and FT3. Higher cumulative days of spraying insecticides and fungicides were significantly associated with reduced FT4 levels after controlling covariates. These findings suggest that pesticides are acting as endocrine disrupters of the hypothalamic-pituitary-thyroid axis.

## Figures and Tables

**Table 1:** Characteristic and risk factors of conventional farmers (*n* = 213) and organic farmers (*n* = 225) at Round 1 (Baseline).

Variables	Conventional Farmers *n* (%)	Organic Farmers *n* (%)	*p*-Value
Age (Year)			
Median (IQR)	52.0 (44.0–58.0)	52.0 (46.0–60.0)	0.084 ^§^
Min-Max	18–69	28–79	
Sex			
Male	158 (74.2)	115 (51.1)	<0.001 ^†^
Female	55 (25.8)	110 (48.9)	
Educational level			
Below elementary	14 (6.6)	4 (1.8)	0.035 ^†^
Elementary	122 (57.3)	125 (55.6)	
High school	72 (33.8)	85 (37.8)	
Bachelor or higher	5 (2.3)	11 (4.9)	
Have Second Job			
Yes	49 (23.0)	128(56.9)	<0.001 ^†^
No	164(77.0)	97(43.1)	
Marital status			
Single	21 (10.1)	13 (6.0)	0.032 ^†^
Married	179 (86.1)	185 (84.9)	
Widowed/divorced	8 (3.8)	20 (9.2)	
BMI (kg/m^2^)			
Normal (<18.49–24.99)	122 (57.5)	167 (74.2)	<0.001 ^†^
Abnormal (≥25.00)	90 (42.5)	58 (25.8)	
Median (IQR)	24.2 (21.2–27.3)	23.0 (20.7–25.1)	0.002 ^§^
Min-Max	16.5–53.0	16.2–37.1	
Total Cholesterol (mg/dL)			
Normal (≤200)	50 (23.5)	123 (55.4)	<0.001 ^†^
Abnormal (>200)	163 (76.5)	99 (44.6)	
LDL (mg/dL)			
Normal (≤100)	21 (10.8)	63 (28.4)	<0.001 ^†^
Abnormal (>100)	173 (89.2)	159 (71.6)	
HDL (mg/dL)			
Normal (≤60)	52 (26.8)	15 (6.8)	<0.001 ^†^
Abnormal (>60)	142 (73.2)	207 (93.2)	
Triglyceride (mg/dL)			
Normal (≤150)	111 (57.2)	143 (64.4)	0.133 ^†^
Abnormal (>150)	83 (42.8)	79 (35.6)	
TSH (μIU/mL)			
Hypo (<0.34)	6 (3.1)	14 (6.4)	0.259 ^‡^
Normal (0.34–5.60)	186 (95.9)	205 (93.2)	
Hyper (>5.60)	2 (1.0)	1 (0.5)	
FT4 (ng/dL)			
Hypo (<0.59)	3 (1.6)	3 (1.4)	1.000 ^‡^
Normal (0.59–1.54)	189 (97.9)	218 (98.2)	
Hyper (>1.54)	1 (0.5)	1 (0.5)	
FT3 (ng/dL)			
Hypo (<0.23)	-	20 (9.0)	<0.001 ^‡^
Normal (0.23–0.49)	192 (99.0)	198 (89.2)	
Hyper (>0.49)	2 (1.0)	4 (1.8)	
T4 (µg/dL)			
Hypo (<6.09)	9 (4.6)	33 (14.9)	0.001 ^‡^
Normal (6.09–12.23)	181 (93.3)	179 (80.6)	
Hyper (>12.23)	4 (2.1)	10 (4.5)	
T3 (ng/mL)			
Hypo (< 0.87)	29 (14.9)	91 (41.0)	<0.001 ^‡^
Normal (0.87–1.78)	161 (83.0)	127 (57.2)	
Hyper (>1.78)	4 (2.1)	4 (1.8)	
Alcohol intake			
Current drinker	136 (63.8)	91 (41)	<0.001 ^†^
Smoking			
Current smoker	59 (26.9)	36 (16.1)	0.006 ^†^
Expense adequacy			
Enough for saving	39 (18.3)	50 (22.2)	0.621 ^‡^
Just enough	100 (46.9)	94 (41.8)	
In debt	73 (34.3)	79 (35.1)	
Stress symptom in the past 2–4 weeks			
Yes	113(53.3)	100(45.0)	0.085 ^†^
Household use of insecticide in the past year			
Yes	191 (89.7)	33 (14.7)	<0.001 ^†^

Statistical analysis using Mann-Whitney U test (§), x^2^ test (†), Fisher Exact test (‡). IQR: Interquartile Range, TSH: thyroid stimulating hormone, FT4: free thyroxine, FT3: free triiodothyronine, T4: thyroxine, T3: triiodothyronine.

**Table 2:** Estimated Marginal Geometric Mean * of Thyroid hormones for four rounds of measurement for conventional and organic farmers.

Parameter	Conventional Farmers Mean (95%CI)	Organic Farmers Mean (95%CI)	*p*-Value from Model F-Test
TSH (µIU/mL)	1.37(1.27–1.48)	1.03(0.96–1.11)	<0.001
FT4 (ng/dL)	0.85(0.83–0.86)	0.87(0.85–0.88)	0.095
FT3 (ng/dL)	0.34(0.34–0.35)	0.32(0.32–0.33)	<0.001
T4 (µg/dL)	7.99(7.76–8.23)	7.56(7.36–7.77)	0.008
T3 (ng/mL)	0.97(0.94–1.00)	0.87(0.85–0.89)	<0.001

* Linear mixed model of Ln thyroid hormone level for all four rounds with a random intercept for subjects by conventional and organic farmers after controlling BMI, gender, and baseline age.

**Table 3:** Estimated Marginal Geometric Mean * of Thyroid hormones for each round of measurement for conventional and organic farmers.

Parameter	Round One Mean (95%CI)	Round Two Mean (95%CI)	Round Three Mean (95%CI)	Round Four Mean (95%CI)
TSH (µIU/mL)				
Conventional farmers	1.28(1.16–1.42) **	1.38(1.23–1.54) **	1.36(1.22–1.51) **	1.48(1.33–1.64) **
Organic farmers	0.90(0.82–0.99)	1.04(0.94–1.15)	1.02(0.93–1.12)	1.20(1.10–1.31)
FT4 (ng/dL)				
Conventional farmers	0.82(0.80–0.85)	0.91(0.89–0.93)	0.82(0.80–0.83) **	0.84(0.82–0.86)
Organic farmers	0.82(0.80–0.84)	0.93(0.90–0.95)	0.88(0.86–0.90)	0.84(0.82–0.85)
FT3 (ng/dL)				
Conventional farmers	0.33(0.32–0.34) **	0.36(0.34–0.37)	0.34(0.33–0.36) **	0.35(0.34–0.36) **
Organic farmers	0.31(0.31–0.32)	0.35(0.34–0.36)	0.32(0.31–0.33)	0.31(0.30–0.32)
T4 (µg/dL)				
Conventional farmers	8.32(8.03–8.63)	7.71(7.50–7.92)	8.08(7.52–8.69) **	7.97(7.68–8.27) **
Organic farmers	8.01(7.76–8.28)	7.80(7.61–8.00)	7.09(6.65–7.56)	7.26(7.02–7.50)
T3 (ng/mL)				
Conventional farmers	1.03(0.99–1.07) **	1.00(0.97–1.04)	0.91(0.85–0.96) **	0.94(0.90–0.97) **
Organic farmers	0.86(0.83–0.90)	1.02(0.99–1.06)	0.75(0.71–0.80)	0.85(0.82–0.88)

* Linear regression of Ln thyroid hormone level for each round with a random intercept for subjects by conventional and organic farmers after controlling BMI, gender, and baseline age. ** Significant differences at *p* < 0.005.

**Table 4:** Self-reported cumulative days of spraying insecticides, herbicides, or fungicides for each consecutive round among conventional farmers.

Cumulative Pesticide Spray Days		Round 1 *n* = 213	Round 2 *n* = 199	Round 3 *n* = 185	Round 4 *n* = 178
Insecticide	Mean (SD)	14 (18)	25 (27)	37 (40)	45 (46)
	Median (IQR)	4 (4–24)	12 (8–36)	20 (12–48)	24 (16–58)
Herbicide	Mean (SD)	7 (7)	17 (13)	25 (16)	34 (23)
	Median (IQR)	4 (4–8)	14 (8–18)	24 (12–32)	29 (18–44)
Fungicide	Mean (SD)	9 (17)	17 (29)	25 (41)	29 (47)
	Median (IQR)	0 (0–8)	8 (0–24)	8 (0–32)	12 (0–38)

SD: Standard Deviation, IQR: Interquartile Range

**Table 5:** Percent change (95% CIs) of thyroid hormones per 10 days of self-reported cumulative pesticide spray days from a linear mixed model * with a random intercept for subjects and days of pesticides sprayed and covariates including BMI, gender, and baseline age.

Thyroid Hormone	Cumulative Insecticide Spray Days	*p*-Value	Cumulative Herbicide Spray Days	*p*-Value	Cumulative Fungicide Spray Days	*p*-Value
TSH	2.92 (1.4, 4.42)	<0.001	4.68 (2.28, 7.07)	<0.001	3.28 (1.6, 4.95)	<0.001
FT3	0.95 (0.51, 1.38)	<0.001	1.72 (0.01, 2.44)	<0.001	0.64 (0.17, 1.11)	0.007
FT4	−0.43 (−0.79, −0.08)	0.017	−0.07 (−0.64, 0.49)	0.802	−0.58 (−0.97, −0.19)	0.004
T3	0.57 (−0.08, 1.22)	0.085	0.18 (−0.89, 1.25)	0.738	0.46 (−0.24, 1.16)	0.197
T4	0.06 (−0.58, 0.70)	0.184	0.42 (−0.63, 1.46)	0.435	−0.11 (−0.81, 0.59)	0.756

* Linear mixed model of Ln thyroid hormone level for all four rounds with a random intercept for subjects by conventional and organic farmers after controlling BMI, gender, and baseline age.
